# Reduction of Polycyclic Aromatic Hydrocarbons (PAHs) in Sesame Oil Using Cellulosic Aerogel

**DOI:** 10.3390/foods10030644

**Published:** 2021-03-18

**Authors:** Do-Yeong Kim, Boram Kim, Han-Seung Shin

**Affiliations:** 1 Research Institute of Biotechnology and Medical Converged Science, Dongguk University-Seoul, 32, Dongguk-ro, Ilsandong-gu, Goyang-si, Gyeonggi-do 10326, Korea; dykimm@dongguk.edu; 2 Department of Food Science and Biotechnology, Dongguk University-Seoul, 32, Dongguk-ro, Ilsandong-gu, Goyang-si, Gyeonggi-do 10326, Korea; marine6535@naver.com

**Keywords:** cellulose, adsorbent, polycyclic aromatic hydrocarbon, sesame seed oil, HPLC/FLD

## Abstract

The effect of cellulosic aerogel treatments used for adsorption of four polycyclic aromatic hydrocarbons (PAHs)—benzo[*a*]anthracene, chrysene, benzo[*b*]fluoranthene, and benzo[*a*]pyrene [B*a*P]—generated during the manufacture of sesame oil was evaluated. In this study, eulalia (*Miscanthus sinensis* var. *purpurascens*)-based cellulosic aerogel (adsorbent) was prepared and used high performance liquid chromatography with fluorescence detection for determination of PAHs in sesame oil. In addition, changes in the sesame oil quality parameters (acid value, peroxide value, color, and fatty acid composition) following cellulosic aerogel treatment were also evaluated. The four PAHs and their total levels decreased in sesame oil samples roasted under different conditions (*p* < 0.05) following treatment with cellulosic aerogel. In particular, highly carcinogenic B*a*P was not detected after treatment with cellulosic aerogel. Moreover, there were no noticeable quality changes in the quality parameters between treated and control samples. It was concluded that eulalia-based cellulosic aerogel proved suitable for the reduction of PAHs from sesame oil and can be used as an eco-friendly adsorbent.

## 1. Introduction

Polycyclic aromatic hydrocarbons (PAHs) are revealed through various routes in the process of manufacturing food. PAHs are a diversified family of more than 100 lipophilic organic contaminants composed of two or more fused aromatic rings [1]. PAHs are derived from the incomplete combustion of organic substances during industrial processes and other human activities [2]. Food processing, such as smoking, grilling, roasting, and toasting, can lead to the formation of high amounts of PAHs [3]. These cooking processes are leading sources of PAHs contamination of oils, seeds, beans, meats, and dairy products [4].

Edible oils are of particular concern regarding PAH intake because of their lipophilic nature [5]. Sesame (*Sesamum indicum* L.) seeds and oil contain a large amount of unsaturated fatty acids, such as linoleic acid and oleic acid, several kinds of sesame lignans, and antioxidants, including tocopherol, sesamin, sesamol, and sesamolin, that may contribute to improved human health [6]. Among its health-promoting activities, sesame oil is reported to modify blood lipid profiles [7] and exhibit antimutagenic activity [8]. PAHs contamination of sesame oil mainly occurs during drying of the pressed solid matter before oil extraction with solvent, roasting, and compression under high temperature.

PAH contamination is a prevailing environmental hazard and a matter of concern for humans and marine ecosystems worldwide, requiring systematic management. PAHs have been categorized by the International Agency for Research on Cancer (IARC) into Group 1 (carcinogenic to humans) and Group 2A (probably carcinogenic to humans) or Group 2B (possibly carcinogenic to humans) [9]. The European Commission (EC) has established maximum levels for benzo[*a*]pyrene (B*a*P), as a group 1 carcinogen, and PAH4, the sum of benzo[*a*]anthracene (B*a*A), chrysene (CHR), benzo[*b*]fluoranthene (B*b*F), and B*a*P, in oils and fats of 2.0 and 10.0 μg/kg, respectively [10].

Although PAH analysis is well-represented in the literature, few techniques have been developed for eliminating PAHs during processing. Previous studies found that the production of PAHs could be reduced during manufacture by using a self-designed apparatus containing washing process, spin-drying before roasting, and ventilation system during roasting or by using adsorbents [11,12]. The existing adsorbents, such as acid clay, activated carbon, and activated charcoal, have been used in edible oil refining. However, these adsorbents were aimed to bleach and deodorize. Due to the insufficient availability of commercial adsorbents for the elimination of PAHs, there is an urgent need to develop new materials. Cellulosic aerogels have received a great deal of recent attention because of their advantages as environmentally friendly adsorbents derived from agricultural by-products. Most ecofriendly cellulose aerogels have been derived from wood substances, and few papers have reported their application for pollutant removal [13,14,15,16,17,18]. Hajeeth et al. [19] extracted cellulose from sisal fiber and grafted it to acrylic acid as an efficient adsorbent for the elimination of heavy metal ions from an aqueous solution. Li and Bai [20] prepared chitosan-cellulose hydrogel beads that displayed high adsorption capacities for Cu.

**Figure 1:**
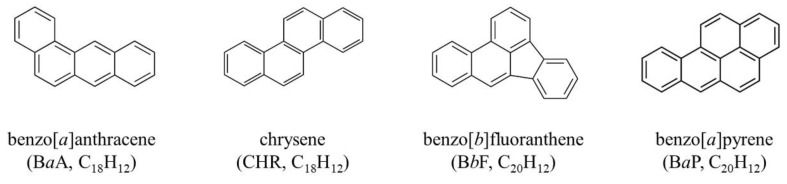
Structure, name, and molecular formula of four polycyclic aromatic hydrocarbons (PAHs) (B*a*A, CHR, B*b*F, and B*a*P).

In this study, cellulosic aerogels derived from *Miscanthus sinensis* (eulalia grass) were applied to the sesame oil processing for the removal of PAHs (B*a*A, CHR, B*b*F, and B*a*P), as shown in Figure 1. Moreover, tests were performed to verify that the quality parameters (acid value [AV], peroxide value [PV], color, and fatty acid composition) of sesame oil were stable after the cellulosic aerogel treatment.

## 2. Materials and Methods

### 2.1. Materials

Analytical grades of solvents, acetonitrile (ACN), methanol, n-hexane, dichloromethane (DCM), and *N,N*-dimethyl formamide (DMFA), were purchased from Burdick & Jackson, Inc. (Muskegon, MI, USA). Sodium sulfate (Na_2_SO_4_) for dehydration was purchased from Junsei Chemical Co., Ltd. (Chuo-ku, Tokyo, Japan). Sep-Pak Florisil cartridges, supplied by Waters Corp. (Milford, MA, USA), were used as solid-phase extraction columns for purification. The PTFE membrane filter (0.45 μm) was obtained from Macherey-Nagel GmbH & Co. KG (Düren, Germany). B*a*A, CHR, B*b*F, and B*a*P standards and 3-methylcholanthrene (3-MC) as an internal standard were obtained from Supelco, Inc. (Bellefonte, PA, USA). Water was obtained from a Milli-Q water purification system (Millipore, Bedford, MA, USA). *Miscanthus sinensis* var. *purpurascens* was provided by the Bioenergy Crop Research Center, National Institute of Crop Science (Jeollabuk-do, Korea). Sesame seeds were acquired from a local market (Seoul, Korea). A standard 37-component fatty acid methyl esters (FAME) mixture was purchased from Sigma−Aldrich Chemical Co. (St. Louis, MO, USA).

### 2.2. Preparation of Cellulosic Aerogel

The cellulosic aerogel was prepared according to previous study [12]. Eulalia (*M. sinensis* var. *purpurascens*) was sieved and shredded to a powder with a size of149 μm. The powder was delignified using a mixture of 99% acetic acid and 30% hydrogen peroxide (1:1, *v*/*v*). It was then put in a water bath (80 °C for 6 h), neutralized by washing, and freeze-dried. The NaOH/urea/H_2_O (7:12:81, *w*/*w*) solution was prepared and cooled at –10 °C. Then delignified powder (3%) was mixed with this solution to synthesize the NaOH/urea-functionalized cellulose-based adsorbent. This solution was added drop-wise into a cylinder filled with methanol, forming beads-type hydrogel. After 12 h, the hydrogels were retrieved, washed with distilled water, subjected to solvent exchange using *tert*-butanol and ethanol, and then freeze-dried.

### 2.3. Preparation of Sesame Seed Oil

**Figure 2:**
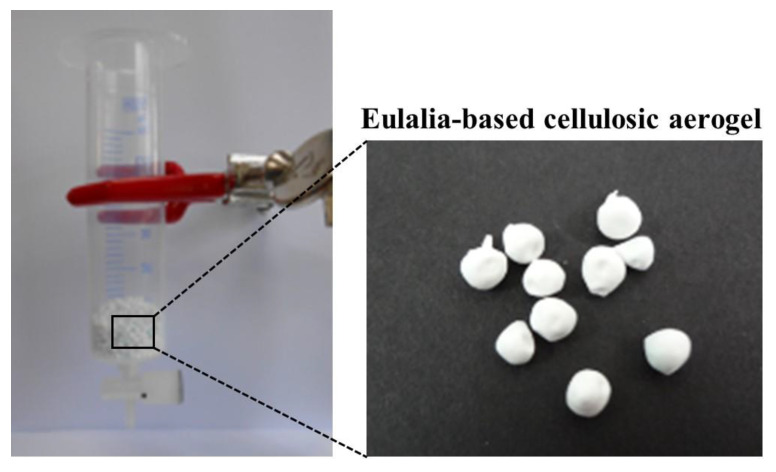
Eulalia-based cellulosic aerogel contained in column.

Sesame seed oil was prepared as described previously [21] with modifications. The sesame seeds were cleaned and then sun-dried (20–24 °C, humidity of 50–70%). Washed sesame seeds were placed in the drum of a hot-air roaster (Gene Cafe CBR-101, Seoul, Korea) and then roasted at 150, 180, 210, and 240 °C for 10, 20, and 30 min. Sesame oil was extracted from roasted sesame seeds using a small expeller (National Eng., NEH-404K, Tokyo, Japan). For treatment with cellulosic aerogel, the syringe-type cartridge was filled with 1% (*w*/*w*, oil volume ratio) of the eulalia-based cellulosic aerogel (Figure 2). The optimal concentration was selected based on the results of our previous studies (Table S1). Sesame oil samples were treated at a rate of 2 to 3 drops per second at room temperature.

### 2.4. Extraction and Purification of PAHs

A 10-g of the sample was weighed and spiked with 1 mL of 5 μg/kg 3-MC, followed by the addition of 100 mL n-hexane. Afterward, n-hexane was extracted with 50, 25, and 25 mL of water: *N,N*-DMFA (1:9, *v*/*v*). The extract was diluted with 1% Na_2_SO_4_ solution (100 mL) and re-extracted with 50, 35, and 35 mL of n-hexane. Pooled n-hexane extracts were washed twice with distilled water (40 mL), dried with 15 g of anhydrous Na_2_SO_4_ and concentrated on a rotary evaporator to 2 mL at 35 °C. The cleaned-up samples were first eluted with 5 mL n-hexane and 15 mL of *n*-hexane−DCM (3:1, *v*/*v*) mixture by a pre-activated solid-phase extraction cartridge. A gentle stream of nitrogen gas at 37 °C was applied to the effluent. The dry residue was redissolved in 1 mL ACN, passed through a 0.45-μm PTFE membrane filter, and then transferred to a high-performance liquid chromatography (HPLC) vial. A 20-μL aliquot of this solution was injected into the HPLC/FLD system.

### 2.5. Analysis of Polycyclic Aromatic Hydrocarbons (PAHs)

PAHs were analyzed by using a Dionex U3000 HPLC system (Sunnyvale, CA, USA) equipped with a Waters 474 fluorescence detector (FLD) (Waters Corp., Milford, MA, USA). Chromatographic separation was performed using aSupelcosil LC-PAH HPLC column (5 µm particle size, 25 cm × 4.6 mm; Supelco, Inc.) at 35 °C. Sample injection volume was 20 µL. The mobile phase consisted of ACN and water at a flow rate of 1.0 mL/min. The elution gradient was 80% ACN in distilled water (*v*/*v*) initially. It was then increased linearly to 100% ACN at 20 min and maintained for 20 min, followed by a linear decrease to 80% ACN for 5 min. The selected pairs of excitation/emission wavelengths were 260/420, 254/390, 260/420, 254/390, and 293/498 nm, for B*a*P, B*a*A, B*b*F, CHR, and 3-MC, respectively.

For validation, PAHs standard mixture was prepared at 0.5, 1, 2, 5, and 10 μg/kg in ACN with 3-MC. The calibration curves were obtained by the injection of standard mixtures (20 μL, in triplicate). The limit of detection (LOD) and limit of quantification (LOQ) were calculated as follows: LOD = 3 × σ/s; LOQ = 10 × σ/s, where s is the slope of the calibration curve; σ is the standard deviation of the y-intercept of the linear regression equation. The recovery rate was determined by analyzing samples spiked with 5 μg/kg of the internal standard. The HPLC/FLD method validation results are presented in Table 1. The coefficient of determination (R^2^) of the calibration curves were high (0.9959–0.9999). The LOD ranged from 0.12 to 0.15 μg/kg, and the LOQ ranged from 0.37 to 0.46 μg/kg. The average recovery of mixed standards was 88.58–92.49%. Values obtained for the validation parameters confirmed the method was suitable for the quantitative analysis of the four PAHs.

### 2.6. Quality Analysis of Sesame Seed Oil

#### 2.6.1. Analysis of Fatty Acid Composition

The fatty acid composition of sesame oil was analyzed by an official method [22] using an Agilent 7890A gas chromatograph (Agilent Technologies, Palo Alto, CA, USA) equipped with a flame ionization detector. The injector and detector temperatures were 250 and 280 °C, respectively. The installed DB-23 column (60 m × 0.25 mm, i.d. × 0.25 μm; J&W Scientific, Folsom, CA, USA) was held at 50 °C for 1 min and programmed to rise to 200 °C at a rate of 25 °C/min, then increase to 230 °C at a rate of 3 °C/min and held at 230 °C for 5 min. The carrier gas (nitrogen) flow rate was 1 mL/min and a split ratio of 20:1. The head pressure was maintained at a constant 230 kPa (33 cm/s at 50 °C). Each FAME in the chromatogram was identified by comparing their retention times with those of the standard 37-component FAME mix.

#### 2.6.2. Analysis of Chemical Properties

The acid value (AV) and peroxide value (PV) of sesame oil were determined by the standard methods (Cd 3d-63 and Cd 8-53, respectively) published by the Association of Official Analytical Chemists (AOCS) [23,24]. Changes in the chemical characteristics of sesame oil with and without cellulosic aerogel (1%, *w*/*w*) treatment were measured.

#### 2.6.3. Chromaticity

Changes in sesame oil color parameters (L*, a*, b*) due to cellulosic aerogel (1%, *w*/*w*) treatment were measured using a Hunter Lab colorimeter (NE 4000, Nippon Denshoku Co., Japan). L* (lightness) ranges from 0 (dark) to 100 (light); a* (redness) ranges from a positive value (red) to a negative value (green); and b* (yellowness) ranges from a positive value (yellow) to a negative value (blue).

### 2.7. Statistical Analysis

All experiments were repeated in triplicate, and One-way analysis of variance (ANOVA) of data was analyzed using SPSS software version 21.0 (SPSS, Inc., Chicago, IL, USA). Significant differences were determined by using Duncan’s multiple range test conducted at a significance level of *p* < 0.05 or by an independent *t*-test at *p* < 0.05.

## 3. Results and Discussion

### 3.1. PAHs Adsorption of Eulalia-Based Cellulosic Aerogel

First, we performed a preliminary test comparing four kinds of cellulose-based adsorbents (tulip wood, pine, eulalia, and radiata pine) and three kinds of commercial adsorbents (active carbon, acid clay, and aluminum silicate). The adsorptive capacity of the adsorbents for BaP in sesame oil is shown in Table S1. Treatment with cellulosic aerogels tended to reduce the yield of edible oil. Increasing the amount of adsorbent (0, 0.3, 0.6, 1, 3, and 5%, *w*/*w*) enhanced BaP removal. Adsorption rates of 19.65, 31.49, 44.76, and 29.11% BaP were achieved following treatment with 1% of the four kinds of cellulosic aerogels (tulip wood, pine, eulalia, and radiata pine, respectively), and the corresponding average BaP concentrations were 10.39, 8.86, 7.14, and 9.17 μg/kg. On the other hand, adsorption rates of 35.90, 10.30, and 17.74% BaP were achieved following treatment with 1% of the three kinds of commercial adsorbents (active carbon, acid clay, and aluminum silicate, respectively), and the corresponding average BaP concentrations were 8.29, 11.60, and 10.64 μg/kg. Although further study is needed, considering the adsorption efficiency, the eulalia-based adsorbent was the most effective in removing BaP from sesame oil. Adsorption rates of 31.56, 36.16, 44.78, 46.74, and 47.32% were determined for 0.3, 0.6, 1, 3, and 5% (*w*/*w*) of the eulalia-based adsorbent, respectively. Above 1% (*w*/*w*), the concentration-dependent efficiency was not significant. Therefore, 1% eulalia-based adsorbent, having the highest adsorptive capacity, was applied to sesame oil manufacturing.

Table 2 shows the concentrations of the individual PAHs and PAH4 in sesame oils under various roasting conditions when treated with or without the cellulosic aerogel. The mean levels of the individual PAHs and PAH4 ranged from 0.17 to 1.80 and 1.69 to 2.97 µg/kg, respectively, in the control sample. In the cellulosic aerogel-treated samples, the mean values of the individual PAHs and PAH4 ranged from not detected (ND) to 0.89 and 0.92 to 1.97 µg/kg, respectively. Considering the PAHs levels following treatment with cellulosic aerogel, the four PAHs and PAH4 contents decreased in all sesame oil samples under the roasting conditions studied (*p* < 0.05), except for CHR after roasting at 210 and 240 °C for 20 min and BbF after roasting at 210 °C for 30 min.

Comparing the change in the PAHs contents as a function of the roasting duration, it was found that the PAHs somewhat increased at 150 and 180 °C as roasting progressed, although the increase was not significant. However, the PAHs contents generally increased significantly at 210 and 240 °C and with roasting duration under these conditions. These data confirmed the influence of temperature and time on the quantity of PAHs formed during the oil processing step. These findings were consistent with Kim and Song [25] who found that a higher heating temperature increases BaP formation significantly.

No samples exceeded the limits recommended by the EC [10] for BaP (2.0 µg/kg) and PAH4 (10 μg/kg) in oils and fats. In particular, BaP, highly carcinogenic, was ND in all but one sample in this study. Therefore, eulalia-based cellulosic aerogel was confirmed to be an effective adsorbent.

In general, adsorption capacity is affected by the intrinsic physicochemical properties of the adsorbate and adsorbents. The cellulose in cellulosic aerogels contains functional groups, such as –OH, –COO, and –COOH, capable of adsorbing PAHs and is thought to form a porous structure (Figure S1) during the process of regeneration. In addition, this process also induced the transformation of cellulose I to cellulose II. This result can explain the change in crystallinity of cellulose and the increase in the adsorption capacity of the cellulosic aerogel.

### 3.2. Quality Properties of Sesame Oil

#### 3.2.1. Analysis of Fatty Acid Content of Sesame Oils

The fatty acid composition of the sesame oil samples roasted under a range of conditions and then treated with and without cellulosic aerogel was analyzed by gas chromatography. Table 3 shows the fatty acid composition of raw and roasted sesame oil. Linoleic acid (C18:2) was a major unsaturated fatty acid, and the main saturated fatty acid was palmitic acid. The fatty acid composition was similar under all roasting conditions: Linoleic acid (C18:2) > oleic acid (C18:1) > palmitic acid (C16:0) > stearic acid (C18:0) > linolenic acid (C18:3n3). Linoleic acid was present at 43.01–45.96%. Oleic acid and palmitic acid were detected in the ranges of 38.81–41.63% and 8.30–9.36%, respectively. These compositions are similar to the results of Kang et al. [26]. After the cellulosic aerogel treatment, the most abundant fatty acid was still linoleic acid (43.01–45.80%). Oleic acid and palmitic acid levels, 37.94–41.05% and 8.00–9.28%, respectively, also remained relatively unchanged. The high content of unsaturated fatty acids is one of the specific characteristics of the sesame oil. Especially, sesame oil is rich in linoleic acid, one of the essential fatty acids, to which is attributed its effectiveness in human nutrition. We confirmed that the cellulosic aerogel selectively adsorbed onto PAHs without significantly affecting the unsaturated fatty acid levels in sesame oil.

#### 3.2.2. Analysis of the Chemical Characteristics

**Figure 3:**
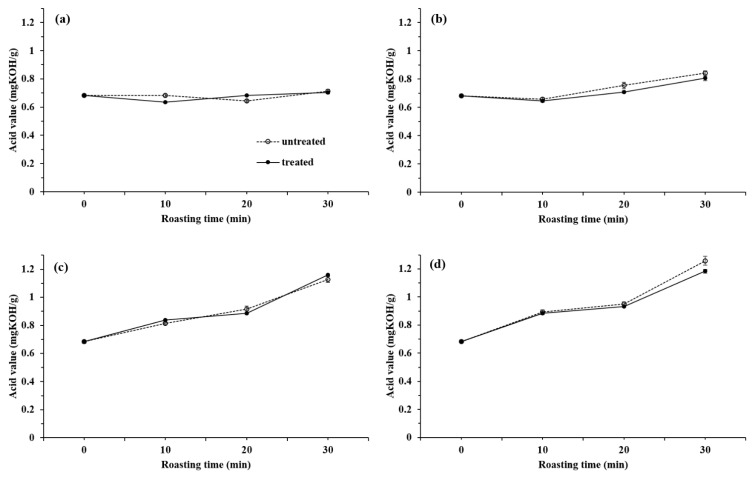
Acid value of sesame oil treated with and without 1% of the eulalia-based cellulosic aerogel under different roasting conditions; 150 °C (**a**), 180 °C (**b**), 210 °C (**c**), and 240 °C (**d**).The PV test was used as the index of the initiation step of oxidation.

**Figure 4:**
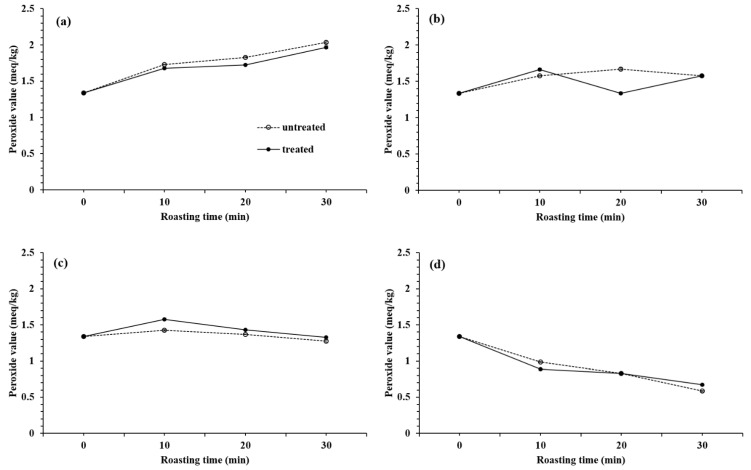
Peroxide value of sesame oil treated with and without 1% of the eulalia-based cellulosic aerogel under different roasting conditions; 150 °C (**a**), 180 °C (**b**), 210 °C (**c**), and 240 °C (**d**).

The AV and PV of sesame oil roasted under various conditions and then treated with and without 1% of the eulalia-based cellulosic aerogel are plotted in Figure 3 and Figure 4. The AV value in the raw sesame oil was 0.68 mg KOH/g. As shown in Figure 3, the average AV of roasted sesame oil was 0.68–1.26, and it tended to increase as the roasting temperature and time increased, especially at the highest temperatures. The AV of sesame oil extracted from sesame seeds roasted at 240 °C (Figure 3d) showed an increasing trend as roasting progressed (0.89, 0.94, and 1.26 at 10, 20, and 30 min, respectively). This result might be due to the oxidation of sesame oil, which produces volatile compounds, such as aldehydes and alkanes. After the cellulosic aerogel treatment, the average AV was estimated as 0.68–1.19 and was not significantly different from the control samples. According to the Codex Standard and the domestic standard (the Korean Food Standards Codex), the AV of sesame oil is stipulated as less than 4.0 [27,28]. The AV of sesame oils treated with and without cellulosic aerogel was compatible with this provision.

In this study, the PV of raw sesame oil was 1.34 meq/kg but increased to an average range of 0.58–12.04 after roasting at low temperature (Figure 4). PV showed an increasing trend during roasting at 150 °C (Figure 4a), perhaps due to the thermal oxidation of the major unsaturated fatty acids in sesame oil, such as linoleic acid and oleic acid. On the other hand, the PV increased initially and decreased with roasting time at 210 °C (Figure 4c), and the PV decreased steadily with roasting time at 240 °C (Figure 4d). This decrease might be caused by the decomposition of peroxide and a similar trend was observed in previous study [29]. After the cellulosic aerogel treatment (Figure 4), the PV ranged of 0.67–2.00 and was not significantly different from the control samples, except for the sample roasted at 180 °C. These oxidation results were similar to the published reports [30,31]. The chemical properties of sesame oil before and after cellulosic aerogel treatment were statistically comparable.

#### 3.2.3. Chromaticity

Color changes in sesame oil due to the roasting conditions were determined by measuring the Hunter L*, a*, and b* values. These parameters are commonly used to show the changes of colors in foods [32]. In the case of sesame oil, L* and a* values tended to decrease, and b* tended to increase as the roasting duration increased. The extent of roasting had a strong influence on L*, a*, and b* values because of the browning reaction. After treating with the adsorbent, L*, a*, and b* values of sesame oil were not significantly different from the control samples (Table 4). From this result, the cellulosic aerogel displayed the adsorption efficiency for PAHs, without causing color changes in sesame oil.

## 4. Conclusions

This study confirmed that cellulosic aerogel treatment might represent an effective strategy to remove PAHs from processed sesame oil. We confirmed the decline in four PAHs when 1% eulalia-based cellulosic aerogel was applied to sesame oil. In addition, fatty acid composition, acid value, peroxide value, and color were measured for physicochemical evaluation of sesame oil. Cellulosic aerogel treatment did not significantly change these properties, even the chemical properties. Recently, industrial manufacturing processes have shifted towards more environmentally friendly processes. Materials are also expected to be recycled, and harmful synthetic materials are being replaced with eco-friendly versions. In the case of PAHs removal by an adsorbent, if food processing is developed to integrate the utilization of natural cellulose-based materials, it has the advantage of minimizing environmental pollution and improving utilization of by-products. Based on the present study, it can be concluded that eulalia-based cellulosic aerogel can be used to reduction of PAHs in sesame oil and have prospects as eco-friendly adsorbents.

## Figures and Tables

**Table 1:** Calibration curve equations, limit of detection (LOD), limit of quantification (LOQ), linearity (R^2^), and % recovery for validation of polycyclic aromatic hydrocarbons (PAHs).

PAH ^1^	Calibration Curve Equation	LOD (μg/kg)	LOQ (μg/kg)	R^2^	Recovery (%)
B*a*A	*y* = 0.1074*x* − 0.0155	0.12	0.37	0.9993	88.58 ± 1.52
CHR	*y* = 0.1086*x* − 0.0263	0.15	0.45	0.9959	91.07 ± 0.85
B*b*F	*y* = 0.1009*x* + 0.003	0.15	0.46	0.9999	89.02 ± 1.29
B*a*P	*y* = 0.3325*x* − 0.0142	0.15	0.45	0.9999	92.49 ± 0.55

^1^ PAH: benzo[*a*]anthracene: B*a*A; chrysene: CHR; benzo[*b*]fluoranthene: B*b*F; benzo[*a*]pyrene: B*a*P.

**Table 2:** Polycyclic aromatic hydrocarbon (PAH) levels (μg/kg) in sesame oil without and with cellulosic aerogel treatment.

		PAH ^1^ (μg/kg)														
Roasting		B*a*A			CHR			B*b*F			B*a*P			PAH4		
Temp. (°C)	Time (min)	Control	Cellulosic Aerogel	*t*-Value	Control	Cellulosic Aerogel	*t*-Value	Control	Cellulosic Aerogel	*t*-Value	Control	Cellulosic Aerogel	*t*-Value	Control	Cellulosic Aerogel	*t*-Value
150	10	0.93 ± 0.01 ^bc^	0.64 ± 0.01 ^c^	35.10 ***	0.41 ± 0.01 ^c^	0.30 ± 0.02 ^d^	10.18 ***	0.18 ± 0.00 ^b^	ND^2^	-	0.18 ± 0.00 ^b^	ND	-	1.71 ± 0.02 ^bB^	0.94 ± 0.01 ^dB^	48.98 ***
	20	0.91 ± 0.02 ^c^	0.64 ± 0.01 ^c^	19.98 ***	0.42 ± 0.01 ^b^	0.27 ± 0.03 ^b^	7.73 **	0.17 ± 0.01 ^d^	ND	-	0.19 ± 0.01 ^b^	ND	-	1.69 ± 0.01 ^dB^	0.92 ± 0.04 ^dB^	36.47 ***
	30	0.95 ± 0.02 ^c^	0.71 ± 0.01 ^d^	20.18 ***	0.44 ± 0.02 ^d^	0.30 ± 0.01 ^d^	10.49 ***	0.18 ± 0.01 ^c^	ND	-	0.19 ± 0.00 ^c^	ND	-	1.77 ± 0.03 ^cA^	1.01 ± 0.01 ^dA^	37.56 ***
180	10	0.91 ± 0.02 ^cB^	0.65 ± 0.01 ^bB^	17.82 ***	0.46 ± 0.02 ^bC^	0.34 ± 0.02 ^cB^	7.97 **	0.19 ± 0.01 ^b^	ND	-	0.19 ± 0.01 ^ab^	ND	-	1.75 ± 0.00 ^bC^	1.00 ± 0.01 ^cC^	92.17 ***
	20	0.92 ± 0.01 ^cB^	0.66 ± 0.02 ^cB^	19.21 ***	0.48 ± 0.01 ^bB^	0.30 ± 0.01 ^bC^	28.47 ***	0.19 ± 0.01 ^c^	0.15 ± 0.01	4.42 *	0.19 ± 0.01 ^b^	ND	-	1.78 ± 0.00 ^cB^	1.12 ± 0.03 ^cB^	40.60 ***
	30	0.97 ± 0.02 ^cA^	0.76 ± 0.02 ^cA^	12.54 ***	0.56 ± 0.01 ^cA^	0.42 ± 0.01 ^cA^	20.66 ***	0.22 ± 0.02 ^c^	0.16 ± 0.01	5.26 **	0.20 ± 0.01 ^c^	ND	-	1.95 ± 0.02 ^bA^	1.34 ± 0.03 ^cA^	27.97 ***
210	10	1.13 ± 0.03 ^aB^	0.79 ± 0.01 ^aB^	16.98 ***	0.47 ± 0.00 ^bB^	0.39 ± 0.01 ^bC^	9.76 ***	0.21 ± 0.01 ^bC^	0.17 ± 0.01	5.35 **	0.20 ± 0.01 ^abB^	ND	-	2.00 ± 0.04 ^aC^	1.35 ± 0.02 ^aC^	24.48 ***
	20	1.13 ± 0.01 ^bB^	0.73 ± 0.02 ^bC^	41.72 ***	0.59 ± 0.08 ^aA^	0.49 ± 0.01 ^aA^	2.12	0.25 ± 0.01 ^bB^	0.20 ± 0.01 ^B^	10.09 ***	0.22 ± 0.02 ^aA^	ND	-	2.20 ± 0.06 ^bB^	1.43 ± 0.00 ^bB^	21.63 **
	30	1.80 ± 0.05 ^aA^	0.89 ± 0.01 ^aA^	30.76 ***	0.62 ± 0.01 ^bA^	0.44 ± 0.01 ^bB^	21.06 ***	0.30 ± 0.04 ^bA^	0.24 ± 0.01 ^A^	2.73	0.24 ± 0.01 ^bA^	ND	-	2.97 ± 0.05 ^aA^	1.58 ± 0.01 ^bA^	43.54 ***
240	10	0.97 ± 0.01 ^bC^	0.63 ± 0.01 ^cC^	34.20 ***	0.53 ± 0.03 ^aC^	0.42 ± 0.01 ^aB^	5.67 *	0.31 ± 0.02 ^aC^	0.20 ± 0.01 ^C^	7.33 **	0.20 ± 0.00 ^aB^	ND	-	2.02 ± 0.04 ^aC^	1.26 ± 0.03 ^bC^	27.48 ***
	20	1.27 ± 0.02 ^aB^	0.98 ± 0.02 ^aA^	18.17 ***	0.61 ± 0.01 ^aB^	0.45 ± 0.03 ^aB^	10.01	0.45 ± 0.00 ^aB^	0.38 ± 0.01 ^B^	10.18 ***	0.24 ± 0.01 ^aA^	ND	-	2.58 ± 0.02 ^aB^	1.82 ± 0.05 ^aB^	22.58 ***
	30	1.30 ± 0.01 ^bA^	0.83 ± 0.02 ^bB^	43.52 ***	0.72 ± 0.01 ^aA^	0.50 ± 0.01 ^aA^	26.30 ***	0.55 ± 0.02 ^aA^	0.41 ± 0.01 ^A^	9.76 ***	0.35 ± 0.02 ^aA^	0.22 ± 0.01	12.95 ***	2.92 ± 0.03 ^aA^	1.97 ± 0.00 ^aA^	46.83 ***

^1^ PAH: benzo[*a*]anthracene: B*a*A; chrysene: CHR; benzo[*b*]fluoranthene: B*b*F; benzo[*a*]pyrene: B*a*P; PAH4: the sum of B*a*A, CHR, B*b*F, and B*a*P; Control: untreated cellulosic aerogel; ^2^ ND: not detected, below the detection limit. ^a–d^ In each line, different letters indicate significant difference (*p* < 0.05) as a function of roasting temperature at the same roasting time and with the same sample by Duncan’s test. ^A–C^ In each line, different letters indicate significant difference (*p* < 0.05) as a function of roasting time at the same roasting temperature and with the same sample by Duncan’s test. Significant differences between the control and cellulosic aerogel-treated groups were analyzed by the *t*-test (* *p* < 0.05, ** *p* < 0.01, and *** *p* < 0.001).

**Table 3:** Fatty acid compositions of sesame oil without (control) and with cellulosic aerogel treatment.

		Fatty Acid (%)									
Roasting		16:0		18:0		18:1		18:2		18:3n3	
Temp. (°C)	Time (min)	Control	Cellulosic Aerogel	Control	Cellulosic Aerogel	Control	Cellulosic Aerogel	Control	Cellulosic Aerogel	Control	Cellulosic Aerogel
150	10	9.30 ± 0.03 ^bA^	9.28 ± 0.00 ^aA^	5.26 ± 0.03 ^bA^	5.26 ± 0.00 ^aA^	39.46 ± 0.05 ^d^	39.45 ± 0.01 ^cA^	45.44 ± 0.03 ^bC^	44.93 ± 0.00 ^aB^	0.44 ± 0.01 ^dC^	0.42 ± 0.00 ^cC^
	20	8.89 ± 0.01 ^cC^	8.37 ± 0.00 ^cC^	5.04 ± 0.01 ^bC^	3.44 ± 0.00 ^dC^	39.43 ± 0.03 ^b^	39.22 ± 0.00 ^cB^	45.96 ± 0.04 ^aA^	44.32 ± 0.00 ^aC^	0.58 ± 0.01 ^aB^	0.53 ± 0.00 ^bA^
	30	9.07 ± 0.01 ^bB^	9.01 ± 0.00 ^aB^	5.21 ± 0.00 ^bB^	4.30 ± 0.01 ^cB^	39.45 ± 0.01 ^b^	39.12 ± 0.01 ^cC^	45.58 ± 0.01 ^bB^	45.76 ± 0.04 ^bA^	0.59 ± 0.01 ^abA^	0.48 ± 0.01 ^cB^
180	10	9.36 ± 0.00 ^aA^	8.29 ± 0.01 ^cC^	5.28 ± 0.01 ^abA^	3.43 ± 0.04 ^dC^	41.63 ± 0.01 ^aA^	41.05 ± 0.01 ^aA^	43.01 ± 0.02 ^dC^	43.61 ± 0.02 ^cC^	0.60 ± 0.01 ^aA^	0.50 ± 0.01 ^aB^
	20	9.08 ± 0.01 ^bB^	8.76 ± 0.00 ^aB^	5.03 ± 0.01 ^bC^	4.66 ± 0.01 ^cB^	39.35 ± 0.05 ^cC^	39.54 ± 0.00 ^bB^	45.84 ± 0.02 ^bB^	44.33 ± 0.00 ^aB^	0.58 ± 0.01 ^aB^	0.57 ± 0.00 ^aA^
	30	9.06 ± 0.01 ^bC^	9.01 ± 0.01 ^aA^	5.04 ± 0.01 ^cB^	4.98 ± 0.01 ^aA^	39.35 ± 0.02 ^cB^	39.31 ± 0.01 ^bC^	45.84 ± 0.00 ^aA^	45.80 ± 0.01 ^aA^	0.59 ± 0.01 ^bB^	0.58 ± 0.01 ^bA^
210	10	9.16 ± 0.01 ^cA^	8.85 ± 0.01 ^bA^	5.21 ± 0.01 ^cB^	5.15 ± 0.01 ^bA^	39.50 ± 0.00 ^cB^	39.32 ± 0.01 ^dB^	45.47 ± 0.01 ^aA^	44.20 ± 0.01 ^bA^	0.55 ± 0.00 ^cB^	0.37 ± 0.01 ^dB^
	20	8.30 ± 0.01 ^dC^	8.00 ± 0.01 ^dC^	4.96 ± 0.00 ^cC^	4.78 ± 0.00 ^bB^	39.41 ± 0.04 ^bC^	39.21 ± 0.01 ^dC^	44.48 ± 0.01 ^cB^	43.27 ± 0.01 ^bC^	0.58 ± 0.01 ^aA^	0.47 ± 0.01 ^cA^
	30	9.08 ± 0.01 ^bB^	8.82 ± 0.01 ^bB^	5.37 ± 0.03 ^aA^	3.61 ± 0.01 ^dC^	40.72 ± 0.01 ^aA^	40.33 ± 0.01 ^aA^	44.35 ± 0.01 ^dC^	43.93 ± 0.01 ^dB^	0.46 ± 0.01 ^cC^	0.38 ± 0.00 ^dB^
240	10	9.36 ± 0.01 ^aA^	8.23 ± 0.00 ^dB^	5.31 ± 0.02 ^aB^	4.37 ± 0.00 ^cC^	40.61 ± 0.01 ^bB^	40.81 ± 0.00 ^bB^	44.03 ± 0.01 ^cB^	43.01 ± 0.01 ^dC^	0.58 ± 0.01 ^bB^	0.47 ± 0.01 ^bB^
	20	9.30 ± 0.01 ^aB^	8.52 ± 0.01 ^bA^	5.72 ± 0.01 ^aA^	4.92 ± 0.05 ^aA^	41.42 ± 0.01 ^aA^	41.05 ± 0.01 ^aA^	43.11 ± 0.01 ^dC^	43.08 ± 0.02 ^cB^	0.34 ± 0.01 ^bC^	0.31 ± 0.01 ^dC^
	30	9.12 ± 0.01 ^aC^	8.22 ± 0.01 ^cB^	4.95 ± 0.00 ^dC^	4.81 ± 0.01 ^bB^	38.81 ± 0.04 ^dC^	37.94 ± 0.00 ^dC^	44.82 ± 0.01 ^cA^	44.75 ± 0.01 ^cA^	0.60 ± 0.00 ^aA^	0.88 ± 0.01 ^aA^
*t*-value		6.01 ***		6.45 ***		1.09 ^NS^		2.54 ^NS^		1.61 ^NS^	

^a–d^ In each line, different letters indicate significant difference (*p* < 0.05) as a function of roasting temperature at the same roasting time and with the same sample by Duncan’s test. ^A–C^ In each line, different letters indicate significant difference (*p* < 0.05) as a function of roasting time at the same roasting temperature and with the same sample by Duncan’s test. Significant differences between the control and cellulosic aerogel-treated groups were analyzed by the *t*-test (*** *p* < 0.001). ^NS^ No significant difference.

**Table 4:** The color (L*, a*, and b*) changes of sesame oil treated with and without 1% of the eulalia-based cellulosic aerogel under different roasting conditions.

		Color					
Roasting		L*		a*		b*	
Temp. (°C)	Time (min)	Control	Cellulosic Aerogel	Control	Cellulosic Aerogel	Control	Cellulosic Aerogel
150	10	94.72 ± 0.01 ^cA^	95.47 ± 0.02 ^bA^	−1.86 ± 0.01 ^aA^	−2.22 ± 0.02 ^bB^	6.94 ± 0.01 ^dC^	6.65 ± 0.01 ^dC^
	20	90.63 ± 0.01 ^aC^	88.84 ± 0.01 ^bC^	−1.90 ± 0.02 ^cB^	−1.64 ± 0.03 ^bA^	8.53 ± 0.01 ^dA^	8.78 ± 0.03 ^dB^
	30	91.37 ± 0.01 ^aB^	90.05 ± 0.06 ^aB^	−1.83 ± 0.02 ^bA^	−1.66 ± 0.02 ^bA^	7.96 ± 0.02 ^dB^	8.89 ± 0.02 ^dA^
180	10	91.16 ± 0.01 ^dA^	92.19 ± 0.05 ^dA^	−1.85 ± 0.02 ^aA^	−1.82 ± 0.01 ^aA^	8.58 ± 0.01 ^cC^	8.49 ± 0.01 ^cC^
	20	89.93 ± 0.03 ^bB^	90.14 ± 0.02 ^aB^	−1.84 ± 0.00 ^bA^	−1.98 ± 0.00 ^cB^	10.09 ± 0.07 ^cB^	9.77 ± 0.02 ^cB^
	30	87.21 ± 0.01 ^bC^	88.51 ± 0.07 ^bC^	−2.22 ± 0.01 ^cB^	−2.12 ± 0.02 ^cC^	11.22 ± 0.03 ^cA^	11.09 ± 0.05 ^cA^
210	10	95.73 ± 0.02 ^aA^	96.06 ± 0.04 ^aA^	−2.83 ± 0.01 ^bC^	−2.92 ± 0.01 ^cC^	10.12 ± 0.01 ^bC^	10.06 ± 0.02 ^bC^
	20	84.26 ± 0.03 ^cB^	85.34 ± 0.02 ^cB^	−2.75 ± 0.02 ^dB^	−2.80 ± 0.02 ^dB^	18.64 ± 0.01 ^bB^	18.31 ± 0.02 ^bB^
	30	45.67 ± 0.02 ^cC^	46.87 ± 0.05 ^cC^	0.06 ± 0.00 ^aA^	0.05 ± 0.01 ^aA^	20.66 ± 0.05 ^bA^	20.08 ± 0.02 ^bA^
240	10	95.10 ± 0.02 ^bA^	94.57 ± 0.02 ^cA^	−3.17 ± 0.01 ^cB^	−3.07 ± 0.05 ^dB^	12.43 ± 0.11 ^aC^	12.15 ± 0.10 ^aC^
	20	81.85 ± 0.04 ^dB^	82.11 ± 0.02 ^dB^	2.60 ± 0.03 ^aA^	2.63 ± 0.02 ^aA^	22.46 ± 0.05 ^aB^	21.10 ± 0.06 ^aB^
	30	20.54 ± 0.02 ^dC^	22.00 ± 0.06 ^dC^	−3.21 ± 0.03 ^dC^	−3.19 ± 0.01 ^dC^	52.59 ± 0.09 ^aA^	52.14 ± 0.10 ^aA^
*t*-value		−0.06 ^NS^		−0.01 ^NS^		0.08 ^NS^	

^a–d^ Values followed by different letters differ significantly (*p* < 0.05) as a function of roasting temperature at the same roasting time and with the same sample by Duncan’s test. ^A–C^ Values followed by different letters differ significantly (*p* < 0.05) as a function of roasting time at the same roasting temperature and with the same sample by Duncan’s test. Significant differences between the control and cellulosic aerogel-treated groups were analyzed by the *t*-test. ^NS^ No significant difference.

## Data Availability

Not applicable.

## Supplementary Materials

The following are available online at https://www.mdpi.com/2304-8158/10/3/644/s1, Figure S1: Scanning electron micrographs of the eulalia-based cellulosic aerogel; surface (a) and inside (b)., Table S1: Effects of cellulose based adsorbents and commercial adsorbents on the levels of Benzo[*a*]pyrene (B*a*P, μg/kg) and adsorption efficiency (%, in parentheses) in sesame oil spiked with 12.93 μg/kg B*a*P.
