# Supplementary material for: Reduction of Polycyclic Aromatic Hydrocarbons (PAHs) in Sesame Oil Using Cellulosic Aerogel
Source: Foods. 2021 Mar 18;10(3):644. doi: 10.3390/foods10030644 (PMC8003230; doi:10.3390/foods10030644)
Supplement: Supplementary file 1 [file foods-10-00644-s001.pdf]

foods MDPI

Table 1. Effects of cellulose based adsorbents and commercial adsorbents on the levels of Benzo[*a*]pyrene (B*a*P, μg/kg) and adsorption efficiency (%, in parentheses) in sesame oil spiked with 12.93 μg/kg B*a*P.

Adsorbents (%, w/w)	Cellulose based adsorbents				Commercial adsorbents		
	**Tulip wood**	**Pine**	**Eulalia**	**Raiata pine**	**Active carbon**	**Acid clay**	**Aluminium silicate**
	**B*a*P (μg/kg)**						
**0**	12.93^{a}	12.93^{a}	12.93^{a}	12.93^{a}	12.93^{a}	12.93^{a}	12.93^{a}
**0.3**	11.12^{b} (14.03)	10.61^{b} (17.95)	8.85^{b} (31.56)	9.91^{b} (23.42)	9.95^{b} (23.11)	12.29^{b} (4.95)	11.37^{b} (12.12)
**0.6**	10.77^{c} (16.74)	9.65^{c} (25.37)	8.26^{c} (36.16)	9.40^{c} (27.33)	9.41^{c} (27.27)	11.99^{c} (7.31)	11.04^{c} (14.67)
**1**	10.39^{d} (19.65)	8.86^{d} (31.49)	7.14^{d} (44.76)	9.17^{c} (29.11)	8.29^{d} (35.90)	11.60^{d} (10.30)	10.64^{d} (17.74)
**3**	9.17^{e} (29.09)	8.15^{e} (37.00)	6.89^{d} (46.74)	8.55^{d} (33.87)	5.00^{e} (61.38)	11.19^{e} (13.47)	10.38^{d} (19.74)
**5**	8.41^{f} (35.02)	7.86^{e} (39.17)	6.81^{d} (47.32)	8.33^{d} (35.57)	4.19^{e} (67.59)	11.24^{e} (13.09)	9.96^{d} (22.97)

$^{a–f}$ Values followed by different letters differ significantly ($p < 0.05$) as a function of the adsorbents concentration (%) at the same adsorbent by Duncan's test.

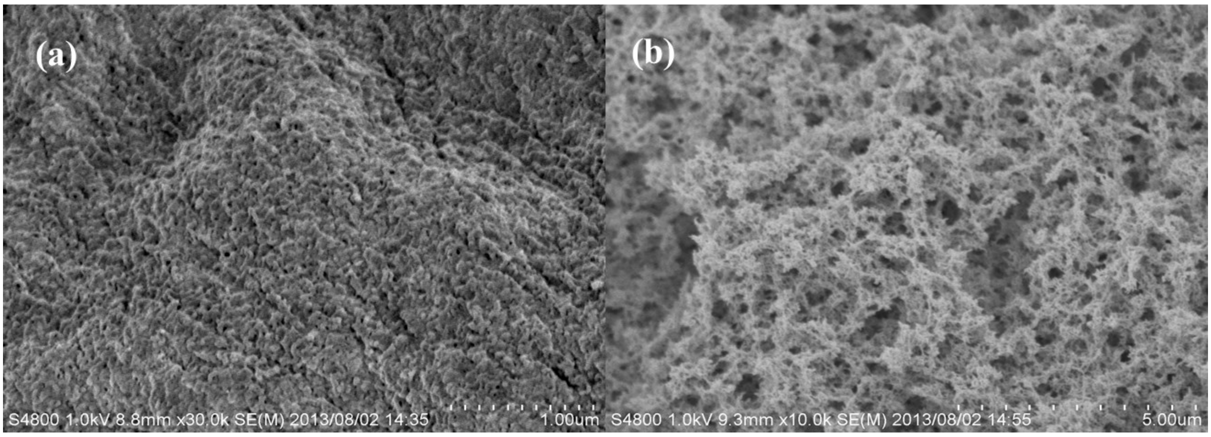

Figure 1. Scanning electron micrographs of the eulalia-based cellulosic aerogel; surface (a) and inside (b).
